# Effect of Adding Alkaline Metal Ions Complexes Rumen Microbiota and Metabolome of Hu Lambs

**DOI:** 10.3390/ani15121816

**Published:** 2025-06-19

**Authors:** Mingyue Li, Chi Ma, Yalin Li, Ziyi An, Yilin Yang, Feng Gao, Changqing Li, Yingchun Liu

**Affiliations:** 1 Institute of Animal Nutrition and Feed, Inner Mongolia Academy of Agricultural and Animal Husbandry Science, Hohhot 010031, China; yue28256@163.com (M.L.); mc1994@emails.imau.edu.cn (C.M.); 2 College of Animal Science, Inner Mongolia Agricultural University, Hohhot 010018, China; liyalin827@163.com (Y.L.); necvk25@163.com (Z.A.); 905963976yyl@gmail.com (Y.Y.); oaa@imau.edu.cn (F.G.); 3 College of Life Sciences, Inner Mongolia Agricultural University, Hohhot 010018, China

**Keywords:** metabolome, microbiome, rumen function, rumen structure

## Abstract

This study aimed to evaluate the effects of studying the effects of the alkali metal ion complexes (AMIC) on the rumen of lambs. Eighteen 3-month-old male Hu lambs (30 ± 2.5 kg) were randomly assigned to three groups (*n* = 6). Dietary treatments were: control group (CG, base diet), group C1 (base diet + 0.15% AMIC), and group C2 (base diet + 0.30% AMIC). After 60 days of feeding, samples were collected for analysis. Compared with CG, rumen weight significantly increased in both C1 and C2 (*p* < 0.05). In C2, average daily gain (ADG), bacterial crude protein (BCP), propionic acid concentration, and rumen papillary length were significantly higher than in CG (*p* < 0.05). Rumen microbiota analysis showed that AMIC supplementation changed the microbial community composition, increasing the relative abundance of fiber-degrading bacteria (e.g., *Prevotellaceae_UCG-001*) and decreasing pathogenic *Proteobacteria*. In particular, rumen papillary length positively correlated with Unclassified *Oscillospiraceae*, *Candidatus Saccharimonas*, and *Unclassified Clostridia vadinBB60 group*. Metabolomic analysis revealed that quercetin 3-*O*-glucuronide levels increased in a dose-dependent manner with higher AMIC. This metabolite positively correlated with *Prevotellaceae_UCG-001* abundance and ADG. At 0.30% AMIC, phospholipids PC(18:0/18:4(6Z,9Z,12Z,15Z)) and PE(18:0/16:1(9Z)) were significantly upregulated, and both positively correlated with *Candidatus Saccharimonas*, *Unclassified Clostridia vadinBB60 group*, and papillary morphology. In summary, AMIC supplementation affected metabolism by modulating the rumen microbiota, thereby promoting energy absorption and growth. The 0.30% AMIC inclusion significantly enhanced rumen papilla growth, increased the absorption area, promoted propionic acid production, reduced the acetic acid to propionic acid ratio, and ultimately improved the growth rate of Hu lambs. Thus, adding 0.30% AMIC was associated with improved growth performance.

## Simple Summary

Alkaline metal ion complexes (AMIC) contain a variety of metal ions, including potassium, sodium, germanium, zinc, and others. These complexes have been shown to play a crucial role in enhancing the growth performance of livestock and poultry, as well as improving the physical immune system. The utilization of this product for livestock feeding has been documented, including cattle, beef cattle, pigs, chickens, and rabbits. However, the impact of AMIC on sheep has not been systematically investigated so far. The present study aimed to elucidate the influence of AMIC supplementation on sheep metabolism. The results showed that *Prevotellaceae-UCG-001* increased the level of quercetin 3-*O*-glucuronide and enhanced the production of propionic acid, while inhibiting pathogenic Proteus mirabilis. Phospholipid mediated nipple elongation expanded the absorption surface area. These synergistic effects increased the average daily weight gain of Hu sheep, reduced feed to weight ratio and acetic acid to propionic acid ratio. In practical production, it can shorten the fattening time of sheep, reduce the risk of acidosis, and achieve sustainable development.

## 1. Introduction

The rumen, a unique digestive organ characterized by a highly diverse microbial community and robust fermentation capabilities, plays a crucial role in the nutrient digestion and metabolic processes of ruminants [1,2]. Bacteria are the most abundant and diverse microorganisms in the rumen of ruminants, accounting for over 90% of the entire microbial community. As a result, they serve as crucial hubs in rumen research [3]. These bacteria exhibit a remarkable degree of diversity, encompassing a diverse array of metabolic types. Rumen bacteria have the potential to impact the host’s health status and gastrointestinal digestion and absorption through their metabolic pathways, thereby playing a crucial role in the host’s health and nutrition [4]. For instance, Butyrivibrio and *Ruminococcus* participate in the digestion and metabolism of nutrients, including cellulose and protein [5]. *Butyrivibrio* mainly regulates the pH value of the rumen and maintains the stability of the rumen environment by synthesizing butyric acid [6]. This stability supports the normal metabolism of other microorganisms, indirectly affecting gastrointestinal digestion and absorption.

Alkaline metal ion complexes (AMIC) contain a diverse range of metal ions that are beneficial for the healthy development of animals, including germanium, sodium, zinc, and potassium, and exhibit biological benefits and therapeutic effects. They can bind to antibacterial proteins such as Lipocalin-2 and Calprotectin, enhancing the organism’s antibacterial capacity [7]. Furthermore, AMIC possesses the capability to alter the diversity and abundance of gastrointestinal microorganisms, reduce the abundance of harmful bacteria in the gastrointestinal tract, promote the reproduction of beneficial bacteria, and enhance digestion and absorption [8,9]. AMIC can enhance the average daily weight gain and total antioxidant capacity of cattle, addressing the issue of blood indicators deviating from the normal range due to heat stress [8,10]. It can alleviate intestinal inflammation caused by weaning stress in piglets, improve daily weight gain, reduce the feed-to-meat ratio, and increase economic benefits [9]. Additionally, AMIC is capable of improving the body weight and feed utilization efficiency of chickens, increasing the villus area in the duodenum, enhancing the level of nutrient absorption, and strengthening intestinal immunity [7]. Furthermore, it can boost the conception rate, 35-day weaning survival rate, and weaning litter weight of rabbits [11]. To date, there have been no reports regarding its impact on the rumen function of meat sheep. Hu sheep is one of the typical breeds of multi-lamb sheep, with excellent traits such as multiple births, strong adaptability, fresh and tender meat, and rich nutrition, which is highly favored by the Chinese sheep industry. Consequently, Hu sheep were selected as the experimental subjects in this experiment. The impact of adding AMIC on rumen structure and function was investigated by measuring the rumen papilla length and width, fermentation parameters, rumen fluid microbial flora, and metabolic indicators. This study conducted comprehensive data analyses across multiple dimensions (growth performance, rumen fermentation parameters, rumen histomorphology, microbiota, and metabolomics) to provide a holistic perspective on the effects of AMIC on Hu sheep. Such a multi-dimensional research approach enhances the comprehensiveness and reliability of the findings.

## 2. Materials and Methods

Ethical approval for all experimental procedures was obtained from the Animal Welfare and Ethics Committee at Inner Mongolia Agricultural University (Approval No. NND 2021105). Animal management and welfare practices strictly adhere to national standards for laboratory animal use, as outlined by the regulatory frameworks of the Chinese Ministry of Science and Technology.

### 2.1. Experimental Animals, Design, and Management

**Figure 1:**
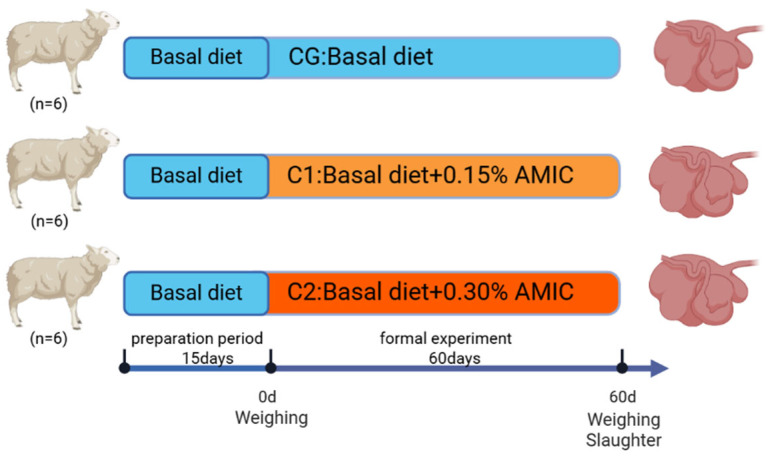
The time flow chart for experimental design and data collection.

The details of the experimental design and the collection of basic data are presented in Figure 1. A total of 18 healthy three-month-old male Hu lambs (body weight: 30 ± 2.5 kg) were stratified by initial BW and randomly allocated to three treatment groups using a completely randomized block design. Each group had six replicates, with one sheep in each replicate. The three groups were the control group (CG), experimental group 1 (C1), and experimental group 2 (C2). The CG group was fed a basic diet, the C1 group had 0.15% alkaline metal ion complexes (AMIC) added to the basic diet, and the C2 group had 0.30% AMIC added to the basic diet. The concentration of AMIC was selected based on our team pre-experimental studies, which showed that at similar doses, AMIC is beneficial for growth performance and rumen health. All diets were prepared as fully mixed pellet feeds.

Throughout the experiment, both food and water were provided ad libitum. The composition and nutritional level of the basic experimental diet are presented in detail in Table 1. AMIC was procured from Beijing Jinnar Biotechnology Co., Ltd., Beijing, China, and its specific composition and content are clearly presented in Table 2.

### 2.2. Growth Performance Determination

At the commencement (day 0) and conclusion (day 60) of the study, each sheep was weighed separately to ascertain the initial and final body weights. The daily feed consumption of each sheep was noted. For every group, calculations were made for the average daily feed intake (ADFI), average daily gain (ADG), and the feed-to-gain ratio (F/G).

### 2.3. Sample Collection

Following humane euthanasia of all experimental sheep, samples of rumen fluid from the dorsal sac were collected. Post-filtration, pH levels were assessed using a portable pH meter. The processed samples were subsequently cryopreserved in liquid nitrogen to ensure sample integrity for downstream analytical procedures.

### 2.4. Rumen Fermentation Parameters

Ammonia nitrogen (NH_3_-N), bacterial protein (BCP) and volatile fatty acid (VFA) were measured as key indicators of rumen fermentation efficiency and microbial activity. NH_3_-N content was measured using an improved colorimetric technique [12], while BCP was determined using an updated colorimetric method at 595 nm [13]. VFA concentration was analyzed using a Shimadzu GC-2014 gas chromatograph (Kyoto, Japan) [14].

### 2.5. Rumen Tissue Morphology

At slaughter, the rumen was removed, rinsed clean, and weighed. A 1 cm piece tissue of rumen from the dorsal sac was collected, paraffin-embedded, sliced (6 μm), stained with H&E, and sealed. Images were captured using a Leica DM750 (Wetzlar, Germany)/Nikon DS-U3 microscope (Tokyo, Japan), and the rumen papilla length, width, and muscle layer thickness were measured using ImageJ software (Version 1.53). Papilla height was measured from the lamina propria base to the keratinized tip, and width at the mid-papilla level.

### 2.6. Rumen Microbiota

Genomic DNA isolation from rumen fluid samples was conducted with the TGuide S96 Magnetic Soil/Stool DNA Kit (Tiangen Biotech, Beijing, China) following standardized protocols. For bacterial community profiling, PCR amplification targeting the hypervariable V3-V4 regions of the 16S rRNA gene was performed using the primer pair 338F (5′-ACTCCTACGGGAGGCAGCA-3′) and 806R (5′-GGACTACHVGGGTWTCTAAT-3′). Following purification, amplicons underwent paired-end sequencing (2 × 250 bp) on an Illumina NovaSeq 6000 system (BMKCloud, Biomarker Technologies Co., Ltd., Beijing, China). Raw sequencing reads underwent quality filtering via Trimmomatic (v0.33), followed by primer sequence removal using Cutadapt (v1.9.1) to generate processed reads. Denoising and chimera elimination were performed using the DADA2 algorithm within QIIME2 (v2020.6), resulting in high-quality non-chimeric sequences for downstream analysis.

### 2.7. Rumen Untargeted Metabolomics

High-resolution mass spectrometric analyses were conducted using a Waters Xevo G2-XS QTOF instrument operated in MSe acquisition mode with MassLynx V4.2 (Milford, MA, USA). During each scan cycle, parallel data collection was achieved by synchronizing low- and high-collision energy channels. Raw spectral data generated through this workflow were subsequently analyzed using Progenesis QI (Milford, MA, USA) for feature detection, spectral alignment, and compound annotation. For metabolites determined by mass spectrometry, they can be identified in the publicly accessible METLIN database and a custom-curated spectral library developed by Biomark (Boise, ID, USA).

### 2.8. Statistical Analysis

Statistical analyses were conducted with one-way ANOVA implemented in SAS (v8.0; SAS Institute, Cary, NC, USA), where significance thresholds were set at *p* < 0.05. Experimental results are presented as the mean values ± standard error of the mean (SEM), and graphical representations were generated using GraphPad Prism (v8.0; GraphPad Software, La Jolla, CA, USA).

## 3. Results

### 3.1. Growth Performance

**Figure 2:**
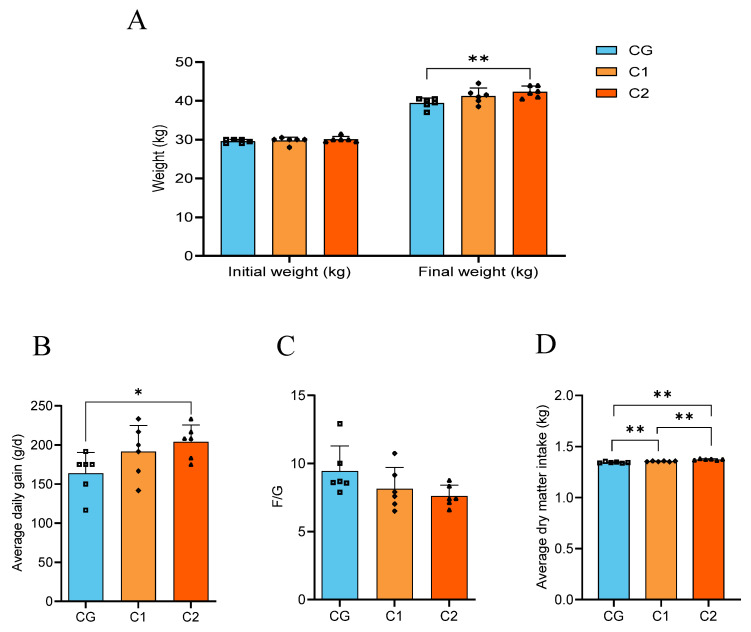
Effect of AMIC on the growth performance of Hu lambs. (**A**) Initial weight and final weight. (**B**) Average daily gain. (**C**) F/G ratio. (**D**) Average dry matter intake. Statistical analysis was performed using ANOVA one-way to compare differences between the two groups. “*” *p* < 0.05, “**” *p* < 0.01.

As shown in Figure 2, compared with the CG group, the average dry matter intake in the C1 and C2 groups was significantly increased (*p* < 0.05). The average daily gain in the C2 group was significantly increased (*p* < 0.05), and the final weight showed an extremely significant increase (*p* < 0.01). The C1 and C2 groups had no significant impact on the feed/gain ratio of Hu lambs, but there was a downward trend (*p* > 0.05).

### 3.2. Rumen Fermentation Parameters

AMIC in the diet of Hu lambs had effects on BCP, propionate, butyrate, and A/P (*p* < 0.05; Table 3), but had no effect on the pH, NH_3_-N, acetate, or total VFA (*p* > 0.05). Compared with the CG group, the C1 group had an increased propionate level (*p* < 0.05), and decreased butyrate and A/P (*p* < 0.05), while the C2 group had significantly increased BCP and propionate contents (*p* < 0.05) and a decreased A/P (*p* < 0.05).

### 3.3. Rumen Slice

**Figure 3:**
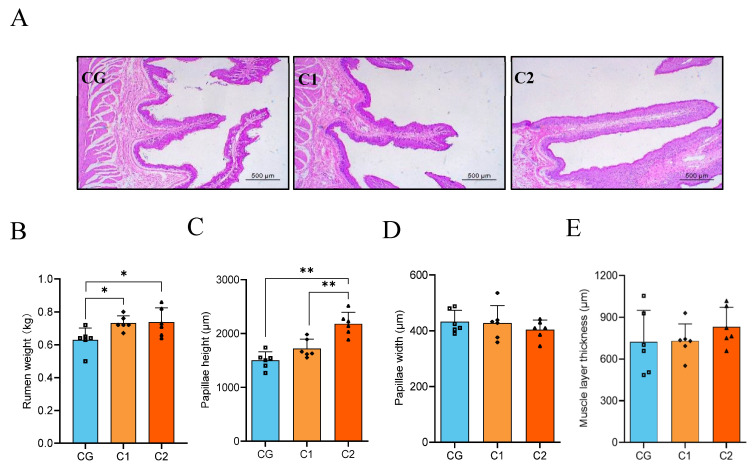
Effect of AMIC on rumen weight and papillae of Hu lambs. (**A**) H&E staining of the rumen papillae. (**B**) Rumen weight. (**C**) Papillae height. (**D**) Papillae width. (**E**) Muscle layer thickness. Statistical analysis was performed using ANOVA one-way to compare differences between the two groups. “*” *p* < 0.05, “**” *p* < 0.01.

AMIC had a significant effect on rumen weight and structure (Figure 3A). Compared with the CG group, the C1 and C2 groups significantly increased rumen weight (*p* < 0.05; Figure 3B). As shown in Figure 3C–E, the C2 group significantly increased papillae height more than the CG and C1 groups (*p* < 0.01), but had no effect on the papillae width and muscle layer thickness (*p* > 0.05).

### 3.4. Rumen Microbiota

**Figure 4:**
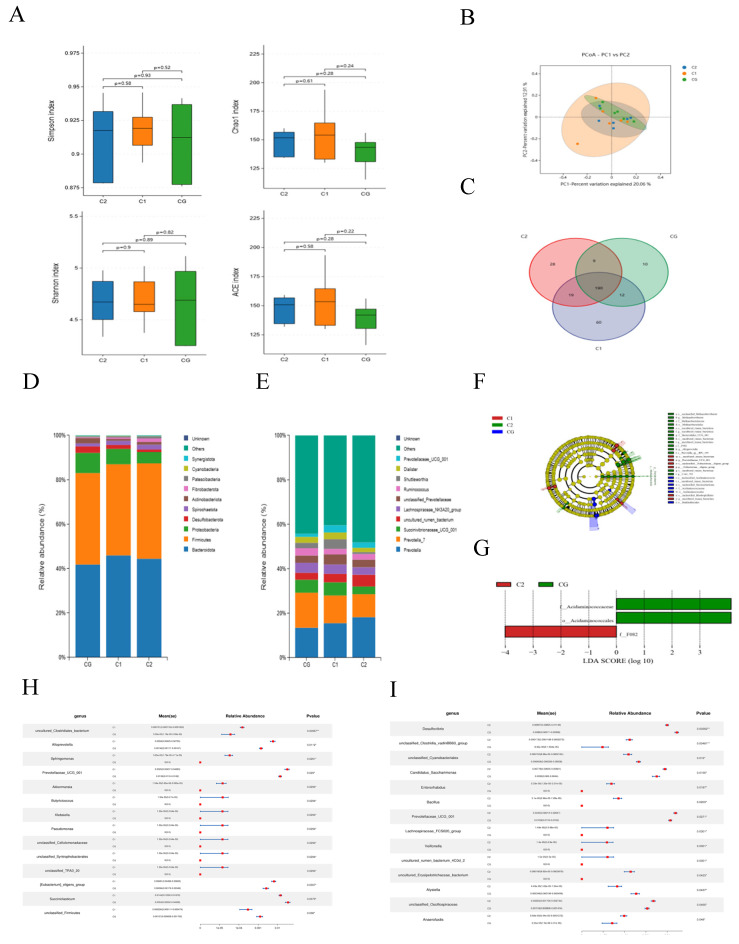
Effect of AMIC on rumen microbiota communities of Hu lambs. (**A**) Alpha diversity indices at the genus level (Shannon index, Simpson index, ACE index, and Chao index) (**B**) PCoA based on genus horizontal Bray–Curtis distance. (**C**) Venn diagram at the genus level. (**D**) Microbial communities bar plot at the phylum. (**E**) Microbial communities bar plot at the genus level. (**F**) LEfSe taxonomy cladogram. The size of the circles is based on relative abundance. (**G**) LEfSe analysis on rumen microbiota. LDA: an LDA score higher than 2 indicated a higher relative abundance in the corresponding group than in other groups. The significantly different species using the nonparametric factorial Kruskal–Wallis rank sum test at a significance level of 0.05. (**H**) At the genus level, Metastats analysis of groups CG and C1. (**I**) At the genus level, Metastats analysis of the CG and C2 groups. Significant correlations are noted by * 0.01 < *p* ≤ 0.05; ** 0.01 < *p* ≤ 0.001.

As shown in Figure 4A, the alpha diversity markers of the Simpson index, Chao 1 index, Shannon index, and ACE index of microbiota showed no significant changes after treatment with AMIC (*p* > 0.05). The result indicated that 20.06% of the variation was captured by axis 1, while axis 2 represents 24.71% of the variation (Figure 4B). As depicted in Figure 4C, the number of unique species in the CG, C1, and C2 groups was 10, 60, and 28, respectively. A total of 202 species were shared between the CG and C1 groups, while 199 species were shared between the CG and C2 groups. Additionally, 209 species were shared between the C1 and C2 groups, among which 190 species were common to all three groups.

The composition analysis of rumen microbiota showed that at the phylum level, *Firmicutes*, *Bacteroidota,* and *Proteobacteria* were the dominant bacteria in the rumen in the CG group, C1 group, and C2 group, while the *Proteobacteria* abundance decreased (*p* > 0.05; Figure 4D). At the genus level, *Prevotella* and *Prevotella_7* were the dominant bacteria in all taxa (Figure 4E). To explore the marker bacteria in rumen fluid among the three groups, microbial community LEfSe analysis was applied. The LEfSe analysis (Figure 4F,G) results showed that in the CG group, *f_Acidaminococcaceae* and *o-Acidaminoccacales* were biomarker bacteria, while after adding alkaline metal ion complexes, the biomarker community in the rumen fluid of the C2 group changed to *f-F082*.

Further analysis showed that at the genus level, compared with the CG group, the abundances of *uncultured_Clostridiales_bacterium*, *Alloprevotella*, *Sphingomonas*, *Prevotellaceae_UCG_001*, *Akkermansia*, *Butyricicoccus*, *Klebsiella*, *Pseudomonas*, *unclassified_Cellulomonadaceae*, *unclassified_Syntrophobacterales*, *unclassified_TRA3_20*, and *[Eubacterium]_eligens_group* in the C1 group significantly increased (*p* < 0.05). The abundances of Succiniclasticum and unclassified_Firmicutes significantly decreased (*p* < 0.05; Figure 4H). In the C2 group, the abundances of *unclassified_Clostridia_vadinBB60_group*, *Candidatus_Saccharimonas*, *Enterorhabdus*, *Bacillus*, *Prevotellaceae_UCG_001*, *Lachnospiraceae_FCS020_group*, *Veillonella*, *uncultured_rumen_bacterium_4C0d_2*, *uncultured_Erysipelotrichaceae_bacterium*, *unclassified_Oscillospiraceae*, and *Anaerofustis* significantly increased (*p* < 0.05). The abundances of *Desulfovibrio*, *unclassified_Cyanobacteriales*, and *Alysiella* significantly decreased (*p* < 0.05; Figure 4I).

### 3.5. Rumen Metabolism

**Figure 5:**
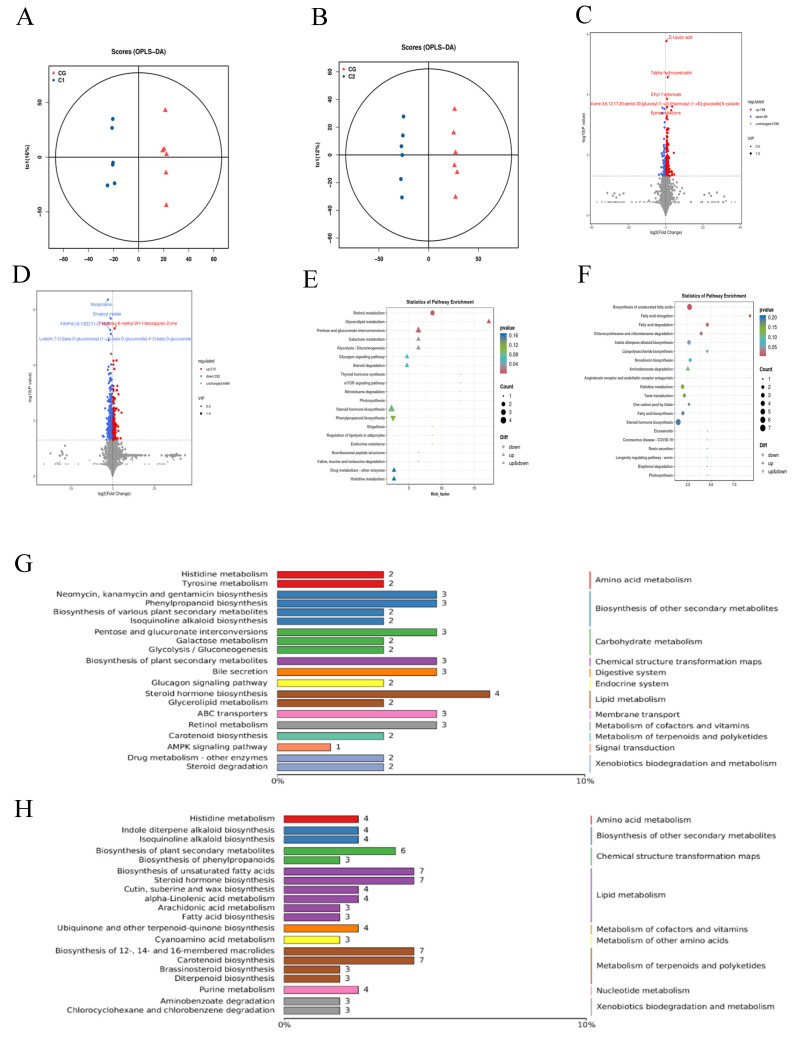
Effect of AMIC on rumen metabolites of Hu lambs. (**A**,**B**) Score plot of orthogonal partial least squares–discriminant analysis (OPLS-DA) for C1 vs. CG and C2 vs. CG, respectively. (**C**,**D**) Volcano plot indicates differential metabolites for C1 vs. CG and C2 vs. CG, respectively. (**E**,**F**) KEGG enrichment analysis of differential metabolites for C1 vs. CG and C2 vs. CG, respectively. The x-axis shows the rich factor of each pathway, the y-axis shows the pathway names, and the color of the dot is the *p* value. A redder color indicates more significant enrichment. The size of the point represents the number of differential metabolites enriched. (**G**,**H**) Summary map of the top 20 items with the most different metabolites for C1 vs. CG and C2 vs. CG, respectively.

The OPLS-DA score plots between CG, C1, and C2 showed significant separation between the groups (Figure 5A,B). After LC-MS analysis, differential metabolites were screened based on VIP > 1 and *p* < 0.05. Compared with the control group, 237 differential metabolites were screened in the C1 group, of which 168 were upregulated and 69 downregulated. In the C2 group, 542 differential metabolites were identified, with 210 upregulated and 332 downregulated (Figure 5C,D).

The metabolic pathways associated with the most differentiated metabolites were identified. The top 20 key pathways of the C1 group are presented in Figure 5E,G. Amino acid metabolism contains two paths: histidine metabolism and tyrosine metabolism. The biosynthesis of other secondary metabolites contains four channels: neomycin, kanamycin, and gentamicin biosynthesis, phenylpropanoid biosynthesis, the biosynthesis of various plant secondary metabolites, and isoquinoline alkaloid biosynthesis. Carbohydrate metabolism contains three channels: pentose and glucuronate interconversions, galactose metabolism, and glycolysis/gluconeogenesis. Chemical structure transformation maps contain the biosynthesis of plant secondary metabolites. The digestive system contains bile secretion. The endocrine system contains the glucagon signaling pathway. Lipid metabolism contains two paths: steroid hormone biosynthesis and glycerolipid metabolism. Membrane transport contains ABC transporters. The metabolism of cofactors and vitamins contains retinol metabolism. The metabolism of terpenoids and polyketides contains carotenoid biosynthesis. Signal transduction contains the AMPK signaling pathway. Xenobiotics biodegradation and metabolism contains two paths: drug metabolism—other enzymes; and steroid degradation. Different metabolites exhibit upregulation across nine key pathways: glycerolipid metabolism, pentose and glucuronate interconversions, galactose metabolism, glycolysis/gluconeogenesis, glucagon signaling pathway, steroid degradation, steroid hormone biosynthesis, drug metabolism—other enzymes, histidine metabolism. Different metabolites exhibit downregulation across one key pathway: phenylpropanoid biosynthesis.

The top 20 key pathways of the C2 group are presented in Figure 5F,H. Amino acid metabolism contains histidine metabolism. Biosynthesis of other secondary metabolites contains two channels: indole diterpene alkaloid biosynthesis and isoquinoline alkaloid biosynthesis. Chemical structure transformation maps contain two channels: biosynthesis of plant secondary metabolites and biosynthesis of phenylpropanoids. Lipid metabolism contains six paths: biosynthesis of unsaturated fatty acids, steroid hormone biosynthesis, cutin, suberine, and wax biosynthesis, alpha-Linolenic acid metabolism, arachidonic acid metabolism, and fatty acid biosynthesis. The metabolism of cofactors and vitamins contains ubiquinone and other terpenoid-quinone biosynthesis. The metabolism of other amino acids contains cyanoamino acid metabolism. The metabolism of terpenoids and polyketides contains four channels: biosynthesis of 12-, 14-, and 16-membered macrolides, carotenoid biosynthesis, brassinosteroid biosynthesis, and diterpenoid biosynthesis. Nucleotide metabolism contains purine metabolism. Xenobiotics biodegradation and metabolism contains two paths: aminobenzoate degradation and chlorocyclohexane and chlorobenzene degradation. Different metabolites exhibit upregulation across one key pathway: aminobenzoate degradation. Different metabolites exhibit downregulation across one key pathway: lipopolysaccharide biosynthesis.

### 3.6. Correlation Analysis

**Figure 6:**
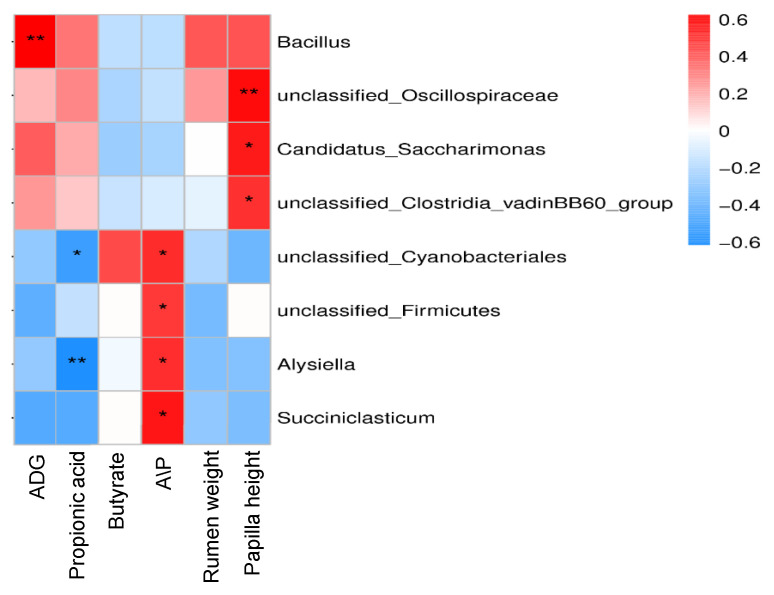
Correlation analysis of rumen microbiota with ADG, Rumen fermentation parameters, Rumen weight and papillae. ADG = average daily gain; A/P = ratio of acetate to propionate. Significant correlations are noted by * 0.01 < *p* ≤ 0.05; ** 0.01 < *p* ≤ 0.001.

As shown in Figure 6, *Bacillus* was positively correlated with ADG (*p* < 0.01). *Unclassified Oscillospiraceae* (*p* < 0.01), *Candidatus Saccharimonas* (*p* < 0.05) and *the unclassified Clostridia vadinBB60 group* (*p* < 0.05) were positively correlated with rumen papilla height. The *unclassified Clostridia vadinBB60 group* was positively correlated with rumen papilla height (*p* < 0.05). *Unclassified Cyanobacteriales* was negatively correlated with propionic acid (*p* < 0.05) and positively correlated with A/P (*p* < 0.05). *Alysiella* was negatively correlated with propionic acid (*p* < 0.01) and positively correlated with A/P (*p* < 0.05). *Unclassified Firmicutes* and *Succiniclasticum* were positively correlated with A/P (*p* < 0.05).

**Figure 7:**
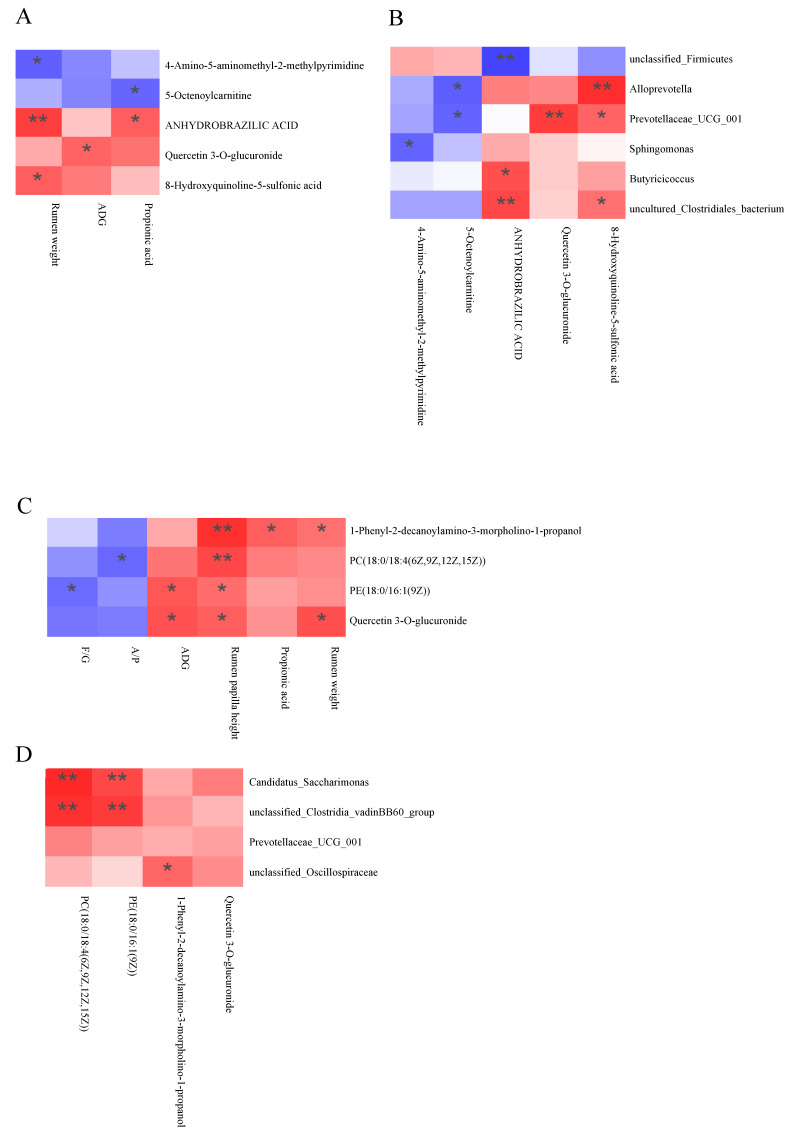
(**A**) Correlation heatmap between C1 metabolites and epigenetic indicators. (**B**) C1 metabolites and microbial correlation heatmap. (**C**) Correlation heatmap between C2 metabolites and epigenetic indicators. (**D**) C2 metabolites and microbial correlation heatmap. Red and blue colors represent positive and negative correlations, respectively, and color gradation indicates the size of the correlation coefficient. Significant correlations are noted by * 0.01 < *p* ≤ 0.05; ** 0.01 < *p* ≤ 0.001 (*n* = 6).

As shown in Figure 7A,B, quercetin 3-*O*-glucuronide exhibited a significant positive correlation with ADG (*p* < 0.05) and *Prevotellaceae_UCG_001* (*p* < 0.01). 5-Octenoylcarnitine showed significant negative correlations with propionic acid, *Alloprevotella*, and *Prevotellaceae_UCG_001* (*p* < 0.05). 4-Amino-5-aminomethyl-2-methylpyrimidine was significantly negatively correlated with rumen weight and *Sphingomonas* (*p* < 0.05).

As illustrated in Figure 7C,D, quercetin 3-*O*-glucuronide displayed a significant positive correlation with ADG and rumen papilla height (*p* < 0.05), but no significant correlation with rumen weight (*p* > 0.05). PC(18:0/18:4(6Z,9Z,12Z,15Z)) demonstrated highly significant positive correlations with rumen papilla height, *Candidatus_Saccharimonas*, and *unclassified_Clostridia_vadinBB60_group* (*p* < 0.01). PE(18:0/16:1(9Z)) was significantly positively correlated with rumen papilla height (*p* < 0.05), and highly significantly correlated with *Candidatus_Saccharimonas* and *unclassified_Clostridia_vadinBB60_group* (*p* < 0.01).

## 4. Discussion

The rumen is a digestive organ unique to ruminants. It is known as the most powerful natural fermentation tank for degrading fibrous substances and plays an extremely crucial role in the nutrition digestion and metabolism of ruminants [15,16]. As a protruding structure on its mucosal surface, rumen papillae are the contact surface between the rumen and external substances. Their healthy development can enhance the body’s ability to resist the entry of pathogens into the rumen and reduce the risk of rumen diseases in sheep [17,18]. The results of this study showed that the addition of AMIC significantly increased the length of rumen papillae.

The state of the rumen papillae is closely related to the growth performance of fattening sheep. Well-developed and functionally normal rumen papillae can promote nutrient absorption and the stability of the rumen internal environment, thereby increasing the daily weight gain and feed conversion rate of fattening sheep [19]. Rumen pH, ammonia nitrogen (NH_3_-N), bacterial crude protein (BCP), and volatile fatty acids (VFA) are important indicators for current research on rumen fermentation, which can reflect the rumen environmental conditions, fermentation type, and fermentation degree [20,21]. The results of this experiment reveal that the pH values and ammonia nitrogen (NH_3_-N) concentrations in each experimental group fall within the normal range. This indicates a robust ruminal buffering capacity and absorption compensation, maintaining homeostasis while enabling beneficial metabolic shifts, thereby demonstrating AMIC’s precision in modulating rumen function without destabilizing core parameters. The content of BCP increases with the increase in AMIC concentration, and the C2 group shows a significant increase. Zn^2+^, as a coenzyme factor, is closely related to protein synthesis. Zn^2+^ may indirectly promote the synthesis of microbial proteins by affecting the activity or stability of microbial protein synthases [22]. BCP is an important source of absorbable protein in the small intestine of sheep, and the increase in its concentration means that sheep have more high-quality protein for the synthesis of muscle tissue [23]. Volatile fatty acids (VFA) can provide up to 80% of the energy for the body and are the main carbon source for the proliferation of rumen microorganisms [24]. This study indicates that adding AMIC to the diet of Hu sheep can increase the concentration of propionic acid and reduce the ratio of acetic acid to propionic acid. VFA is transported in the rumen epithelium through various pathways, including Na^+^/H^+^ exchange. Na^+^ promotes Na^+^/H^+^ exchange, enhances VFA absorption, provides energy sources for ruminants, and also provides energy sources for rumen epithelial cells [25]. In addition, Froetschel et al. [26] found that supplementing zinc in beef cattle feed can increase the molar concentration of propionic acid and reduce the ratio of acetic acid to propionic acid. The addition of AMIC can increase the concentration of propionic acid, reduce the ratio of acetic acid to propionic acid, and promote the length of the rumen papillae, which is a result of the combined action of Na^+^ and Zn^2+^. Propionic acid is the main gluconeogenic precursor substance in ruminants. It can be converted into glucose through gluconeogenesis in the liver. The body may utilize glucose more for energy metabolism, providing sufficient energy for sheep to support muscle growth and fat deposition and improving the weight gain and feed utilization efficiency of sheep [27,28]. The results showed that adding 0.15% and 0.30% AMIC increased daily weight gain by 27.78 g and 40.00 g, respectively, and reduced the feed-to-gain ratio. Cheng et al. [29] proved that the addition of AMIC could improve the body weight and daily weight gain of calves, which is similar to the results of this experiment. The sheep in the C2 group not only had a significant increase in the content of propionic acid in the rumen, but also had an increase in the length of the rumen papillae, which enlarged the absorption area of the rumen for propionic acid. This helps the body to absorb propionic acid better, utilize glucose more for energy metabolism, reduce the dependence on fat mobilization, and thus improve the energy utilization efficiency. This also explains the reason for the downregulation of the lipolysis pathway in the C2 group.

The rumen papillae directly affect the growth environment of rumen microorganisms. Healthy rumen papillae can provide attachment sites for microorganisms, and rumen microorganisms can tightly attach to the surface of the papillae for growth and reproduction [30]. The present study investigated the effects of AMIC on the rumen microbiota of Hu lambs, revealing nuanced shifts in microbial composition and diversity despite the absence of significant changes in α-diversity indices (Simpson, Chao 1, Shannon, and ACE). The results of PCoA and the Veen plots of three groups of species also confirm this viewpoint. At the phylum level, *Firmicutes* and *Bacteroidota* remained dominant across all groups, *Bacteroidota* mainly degrade non-fibrous carbohydrates in the diet, while *Firmicutes* mainly degrade cellulose in the diet, secreting various metabolic enzymes to promote energy absorption and fat deposition in ruminants [17,31]. *Proteobacteria* are sensitive to feed and environmental factors and accumulate a large number of harmful bacteria [32]. Their proliferation and enrichment reflect an ecological imbalance, unstable rumen microbial community structure, or host disease status [33]. The abundance of *Proteobacteria* exhibited a progressive decline across groups, with the CG, C1, and C2 groups showing mean relative abundances of 9.07%, 6.67%, and 5.00%. Zinc acts as a complex antioxidant by participating in superoxide dismutase (SOD) reactions, stabilizing cell membranes, and inhibiting lipid peroxidation. Zn^2+^ maintains the redox balance of pathogens by reducing ROS levels in their cells, thereby inhibiting their growth [34]. Meanwhile, germanium (Ge) is a trace element of various organic substances that has been discovered, with a wide range of activities including antioxidant and anticancer effects [35]. At the genus level, *Prevotella* is the genus with the highest abundance in the rumen and is considered to play an important role in the rumen ecosystem [36]. It can utilize various substrates such as starch, monosaccharides, and other non-cellulosic polysaccharides as energy sources to produce succinic acid [37]. The results of this experiment showed that *Prevotella* and *Prevotella_7* are three dominant microbial genera in the rumen of Hu sheep. The average abundance of *Prevotella* in group CG was 13.75%, that in group C1 was 15.51%, and that in group C2 was 18.48%. This may be because Na^+^ maintains osmotic pressure, optimizes pH, drives energy metabolism, and collaborates with HCO_3_^−^ and Zn^2+^ ions to create a suitable rumen environment for *Prevotella* growth [38]. It indicates that the addition of AMIC can, to a certain extent, increase the abundance of relevant bacterial genera in the rumen of Hu sheep that have the ability to degrade xylan, pectin, and proteins [39,40]. Additionally, the reduction in *Desulfovibrio* in C2 may decrease hydrogen sulfide production, thereby potentially alleviating rumen dysbiosis [41]. LEfSe analysis identified group-specific biomarker taxa, revealing functional shifts induced by AMIC supplementation. In the CG group, the enrichment of *f_Acidaminococcaceae* suggested enhanced amino acid fermentation, potentially associated with inefficient nitrogen utilization. Conversely, C2 exhibited *f_F082* (*Lachnospiraceae* family) as the key biomarker—a butyrate-producing taxon implicated in fiber degradation and epithelial health [42]. These biomarker shifts demonstrate that AMIC redirects microbial metabolism from protein catabolism to fiber saccharification, thereby enhancing energy harvest and rumen development.

Further research has found that *Candidatus Saccharimonas*, the *unclassified Clostridia vadinBB60 group*, and *unclassified Oscillospiraceae* were significantly positively correlated with the length of the rumen papillae. *Candidatus Saccharimonas* plays a crucial role in regulating the host immune response. It is positively correlated with various immune cells (such as CD4+ T cells) and may play a regulatory role in the immune system [43]. The *Clostridia vadinBB60 group* is positively correlated with the host’s immunomodulatory cells (such as regulatory T cells, Tregs) and may alleviate the inflammatory response by regulating the immune system [43]. The abundance of *Oscillospiraceae* is positively correlated with the diversity of the gut microbiota and is an important indicator for assessing the health status of the microbiota [44]. These three play roles in immunomodulation, metabolic functions, and microbiota diversity, which is beneficial for maintaining rumen health, thus promoting rumen papillae growth.

There is a dynamic correlation between metabolites and microorganisms, and studying metabolites is helpful for a better understanding of the mechanism of rumen development [39]. Further exploration of metabolites led to the identification of PC(18:0/18:4(6Z,9Z,12Z,15Z)) and PE(18:0/16:1(9Z)) as metabolites related to *Candidatus Saccharimonas* and the *unclassified Clostridia vadinBB60 group*. PC(18:0/18:4(6Z,9Z,12Z,15Z)) is a phosphatidylcholine (PC) and PE(18:0/16:1(9Z)) is a phosphatidylethanolamine (PE), both of which belong to the glycerophospholipid class. Phosphatidylcholine is an important component of cell membranes and is involved in processes such as the maintenance of cell structure, signal transduction, and lipid metabolism [45]. In addition, both are also correlated with the average daily gain (ADG), propionate, and the acetate-to-propionate ratio. It is hypothesized that these two substances affect the growth and differentiation of rumen epithelial cells, maintain the integrity and function of rumen epithelial cells, influence the health and development of rumen papillae, expand the rumen absorption area, increase the propionate content, and decrease the acetate-to-propionate ratio, thereby increasing ADG.

In addition, it was also found that quercetin 3-*O*-glucuronide was significantly and positively correlated with both the abundance of *Prevotellaceae_UCG_001* and the average daily gain (ADG). Quercetin 3-*O*-glucuronide, a flavonoid compound, has antioxidant, anti-inflammatory, and antibacterial properties. It may promote energy utilization by regulating the rumen microbiota and host metabolism [46,47]. It is speculated that Zn^2+^ may provide a favorable environment for the synthesis and accumulation of quercetin 3-*O*-glucuronide by protecting metabolic enzyme activity and reducing oxidative damage. These results suggest that the addition of AMIC may affect carbohydrate metabolism by altering the abundance of *Prevotellaceae_UCG_001* and increasing the content of quercetin 3-*O*-glucuronide, thus promoting the absorption of energy by organisms and facilitating their growth.

## 5. Conclusions

Dietary supplementation with 0.30% alkaline metal ion complex (AMIC) optimizes rumen function in Hu lambs by modulating microbiome–metabolome interactions: The elevation of quercetin 3-*O*-glucuronide driven by *Prevotellaceae_UCG-001* enhances propionate production while suppressing pathogenic *Proteobacteria*, and phospholipid-mediated papillary elongation expands the absorptive surface area. These synergistic effects yield commercially significant outcomes: higher average daily gain, improved feed efficiency, and reduced acetate/propionate ratios. This provides a science-backed strategy for precision finishing systems. By implementing 0.30% AMIC in intensive production, finishing cycles can be shortened and the acidosis risk reduced, ultimately leading to sustainable sheep meat production.

## Figures and Tables

**Table 1:** Composition of the basal diet (as-fed basis).

Item	Ingredient, %	Nutrient Composition	Content, g/kg
Corn	29.00	DE (MJ/kg)	11.18
Melon seed shells	16.00	Dry matter (%)	89.11
Corn germ meaL-so1	14.50	Crude ash (%)	9.12
Soyabean meal	6.00	Crude Protein (%)	19.71
Cottonseed meal	5.50	Crude fat (%)	12.90
Black flour	5.00	Neutral detergent fiber, NDF (%)	32.68
DDGS	4.00	Acid detergent fiber, ADF (%)	13.77
Feed material, sprayed corn bran	3.00	Acid detergent lignin, ADL (%)	2.28
Jujube powder	3.00	Calcium (%)	1.93
Phragmites australis	3.00	Total phosphorus (%)	0.43
Rice protein meal	2.00		
Molasses	2.00		
Bentonite	1.50		
Stone dust	1.30		
Soybean germ powder	1.00		
Calcium sulfate	1.00		
Salt	0.70		
Premix	1.50		
Total	100		

Premix supplied per kilogram diet: vitamin A, 10,000 IU; vitamin D, 1500 IU; vitamin E, 80 IU; Cu, 20 mg; Fe, 40 mg; Zn, 25 mg; Mn, 30 mg; I, 1.5 mg; Co, 0.7 mg; and Se, 0.6 mg. DE is the calculated value, the others are measured values.

**Table 2:** Ionic concentration in AMIC.

Ions	Calculate Concentration (mg/kg)
Na^+^	27,482.00
K^+^	25,103.00
Zn^2+^	5.20
Ge^4+^	0.13
HCO_3_^−^	36,218.00

All values are calculated concentrations.

**Table 3:** Effect of AMIC on rumen fermentation parameters of Hu lambs.

Item	CG	C1	C2	SEM	*p*-Value
pH	6.01	6.07	6.12	0.04	0.1707
NH_3_-N (mg/dL)	12.32	12.94	12.80	1.32	0.9422
BCP (mg/dL)	28.78 ^a^	30.25 ^ab^	30.87 ^b^	0.63	0.0327
Acetate (mmol/L)	38.03	38.12	40.84	2.26	0.6143
Propionate (mmol/L)	21.01 ^a^	27.41 ^b^	26.89 ^b^	1.63	0.0248
Butyrate (mmol/L)	10.23 ^a^	7.97 ^b^	8.97 ^ab^	0.67	0.0303
Acetate/Propionate	1.90 ^a^	1.40 ^b^	1.52 ^b^	0.12	0.0231
Total VFA (mmol/L)	69.26	73.50	76.70	3.73	0.3920

Values with the same superscript letters indicate no significant difference (*p* > 0.05), adjacent letters denote significant differences (*p* < 0.05), and non-adjacent letters signify extremely significant differences (*p* < 0.01).

## Data Availability

The authors confirm that the data supporting the findings of this study are available within the article.
